# An Architecture for Reliable Transportation of Delicate Goods

**DOI:** 10.3390/s21082645

**Published:** 2021-04-09

**Authors:** Paulo Matos, José Rufino, Rui Lopes

**Affiliations:** 1 Instituto Politécnico de Bragança, Campus de Santa Apolónia, 5300-253 Bragança, Portugal; 2 Research Centre in Digitalization and Intelligent Robotics, Instituto Politécnico de Bragança, Campus de Santa Apolónia, 5300-253 Bragança, Portugal; rufino@ipb.pt (J.R.); rlopes@ipb.pt (R.L.)

**Keywords:** transportation, delicate goods, IoT, ultra-low power, BLE, blockchain

## Abstract

Adequate conditions are critical to avoiding damage or degradation of products during transportation, especially in the case of delicate goods like food products, live animals, precision machinery or art items, among others. The damages are not always readily identified: sometimes they are only detected several days or weeks after the merchandise has been delivered. Moreover, it may be hard to assess if the problems resulted from the transport conditions, and it may be even harder to prove it, making it difficult to determine and assign responsibilities. Also, transport is a global business, typically involving different companies and means (truck, train, plane, ship, …). Usually, customers hire the service to a single commercial entity, but the service is performed by several companies, like transporters, stockists and dispatchers. To know whether the transport requirements are fulfilled or not is thus essential to assessing responsibilities and encouraging compliance by all the players in the process. In this paper, the authors propose an architecture that allows certifying, in an exempt manner, the conditions under which the transport of sensitive goods are carried out. In case of compliance, it protects the entities of the transport chain and ensures the customer that the merchandise has not been subject to conditions that may have affected its integrity or quality. If problems are detected, it allows to identify the non-compliant players and to assign responsibilities. The solution is based on ultra-low-power, low-cost devices (equipped with several sensors, a real-time clock, and Bluetooth Low Energy services), a mobile application and several cloud services (including a Coordinated Universal Time service).

## 1. Introduction

Freight transport is one of the world’s main economic activities and an essential pillar of many other activities. It has been growing around 3% per year [1]. The service provided is based on the major premise of transporting goods between two locations, charging for this service. The scale of this activity is so huge that it gives rise to very distinct contexts and requirements, reaching the point of supporting intercontinental commercial circuits with deadlines and periodicities that are quantified in hours.

Besides the delivery time and guarantee of delivery, other requirements are now critical, namely the transport conditions, whether as a result of the quality requirements of the end customer, or simply as a way of making certain transactions (business) feasible, which without the guarantee of the transport conditions are not viable.

Current practice assumes that the service provider complies with transport conditions. However, even when such conditions concern only the deadlines (which most of time are effectively accomplished), it is also accepted that the service is not 100% guaranteed.

When transport also lacks controlled conditions, such as temperature or humidity, the confirmation that the service was provided as agreed is often based on an empirical verification of the status of the goods after the product delivery (or, at most, in audits carried out, with some regularity, on the transport service).

In most situations, the guarantee that the service is provided as agreed is based on a commodity insurance which, in case of loss or degradation of the merchandise, safeguards the customer. But that does not avoid undesirable costs, either for the insurance itself, or for compensation when needed. Further costs can be added, namely those related to non-compliance with contractual supply obligations or stock failure, which can have repercussions in terms of the corporate image of those involved or even lead to lost business opportunities.

In addition, there may be environmental costs: a commodity that required a certain amount of resources to be produced and transported, once spoiled/unusable turns into waste, most likely with a direct environmental impact or extra costs in order to be recycled. The principle that there is insurance that protects the target in case of damage or loss of goods, particularly as a result of not safeguarding the transport conditions, is antagonistic with the current social requirements that foster a sustainable and circular economy, whose main premise is to avoid the production of waste.

Moreover, transport conditions are particularly relevant for delicate goods [2,3,4]. This is the case of food, such as meat, fish, fruit or vegetables, which must be transported under very well-defined (and often regulated) conditions of temperature, humidity, or even ventilation. This type of merchandise is tradable on a global scale, whether in fresh, frozen or other forms. Brazil, for example, is one of the major world exporters of animal protein (namely of bovine origin [5]) supplying markets as far away as China or Russia, with long and distant routes (mostly done by sea). In this case, the origin of the meat, who produces it, and what certifications it has, are some of the important factors for the valorization of the product, and also for the sustainability of the business. They demand investment and time, aiming to convince and win consumer confidence, which is not an easy task. However, it is not enough to produce the best product if the transport conditions jeopardize this quality and recognition.

The transport of delicate goods quite normally involves road transport with refrigeration chambers, often operated by subcontractors or individual workers, who do not respond directly to those who hire the transport service and, as such, are less concerned with the quality of the provided service and more with their own profit. This work has been carried out in close partnership with some transport companies operating in the Brazilian market, themselves service providers, but also subcontractors, who pointed out that the reduced negotiating capacity and the high competition leads carriers to lower prices and very controlled profit margins, without room for unforeseen circumstances and forcing minimization of expenses. Sometimes, this goes beyond the acceptable limits, as when the cooling system is turned off to reduce fuel consumption, or avoiding resorting to warehouses where the merchandise can be kept in due condition, especially during overnight breaks. Such carelessness easily creates conditions to degrade the product or even allow its bacteriological contamination. Being that this a reality, supported by evidence reported in several studies [6,7,8,9], neither the producers nor the consumers can be absolutely sure that the transport is carried out in due conditions. Moreover, without a direct responsibility, the producer ends up selling a product of inferior quality or even out of conditions for consumption.

However, the transport of delicate goods relates to other situations, such as the transport of live animals, involving two major segments: animals for slaughter (for human consumption or other purposes), and pets or animals for competition. In either case, transport conditions can have consequences that are not immediately detectable. For example, an animal can be delivered alive, apparently in good health, but after a few days or weeks passes away or becomes seriously ill. Thus, an animal whose health conditions prevented slaughter for human consumption (or even for any other type of consumption), loses much (if not all) of its commercial value. Or, if the animal is a pet or a competitor, very high emotional/economical losses may be at stake. Ascertaining the causes for such situations is difficult enough, but if the suspicions are due to non-compliance with transport conditions, then it is practically impossible to prove it.

There is also the case of merchandise, such as art or archaeological pieces, sometimes priceless and irreplaceable. Or technical equipment of high precision, or extremely delicate, which cannot be subjected to falls, sudden movements or inclinations. These, being undetectable in the merchandise, can jeopardize their correct operation.

On the other hand, examples exist where proper conditions are ensured a priori. For example, when transporting organs for transplantation, care is taken to pack the organ in a suitable container for that purpose, with controlled temperature and humidity. The transport operator is essentially responsible for not subjecting the packaging to violent displacements and delivering it on time. In any case, this type of transport is done under very special conditions, rarely using conventional commercial services.

Another example, emerging from the present scenario of the COVID-19 pandemic, is the logistics associated with two of the available vaccines, from Pfizer and BioNTech [10,11,12], that must be kept at −70 °C during transport and storage. Since there is an evident lack of vaccines on the market, and since it is urgent to vaccinate as many people as possible, vaccines are a very critical resource that cannot be wasted. The goal of vaccinating as many people as possible in the shortest amount of time has triggered some of the largest logistical operations ever carried out in order to guarantee that the vaccines can be applied to hundreds of millions of people (including health care professionals on thousands of hospitals and health centers). The viability of these logistical operations, or at least the speed of implementation, largely depends on the use of existing logistical resources, adapted to the new requirements. Since the vaccines leave the factory and people are inoculated, they will have to pass through many actors and operational contexts. Who can effectively ensure that they arrive properly?

In short, it is fundamental to provide transport services of better quality and with more guarantees than simply meeting the delivery deadline. The solution naturally depends on the conditions created by the transporters and remainder logistics operators, but it is also up to the market to demand more effective guarantees that the service is provided under the agreed conditions. Producers, carriers (including sub-contractors) and end costumers would all benefit from it.

The authors present in this article an open and generic architecture and protocols, designed to guarantee the quality of the transportation service for all the involved, including the customer. The architecture was designed to be adopted by any carrier that wants to provide guarantee of the conditions agreed for the transportation of the merchandise, without requiring significant changes to the companies’ operational model, with a reduced investment and a low operational cost.

The remainder of this article is organized as follows: the most novel solutions available on the market that can be compared with this work are presented in Section 2; the technical options that are available (with a focus on their advantages and limitations) are discussed in Section 3; the high level architecture, the IoT devices that support it and the topologies idealized to enable a widespread use of the solution are presented in Section 4; the technical details of the protocols, including the procedures for the aggregation and disaggregation of merchandise, are described in Section 5; an overall discussion of this proposal, together with the conclusions and directions for future work, are presented in the final section.

## 2. Market Analysis

Ambrosus [13] is a blockchain-powered IoT network [14,15] for food and pharmaceutical enterprises, enabling secure and frictionless dialogue between sensors, distributed ledgers and databases to optimize supply chain visibility and quality assurance. The enterprise behind it idealized a commercial exploration model and used the Ethereum blockchain [16,17,18] to support their own version of a blockchain-IoT platform, the AMB-NET. For that, it provides several SDKs that can be used by third parties (Ambrosus customers) to initiate smart contracts [19,20]. These track the entire lifecycle of a product, providing an immutable and tamper-proof record of the collected data and the conditions agreed for the transportation of the merchandise.

Though sharing some of the goals of the architecture proposed by the authors, AMB-NET is a possible implementation of only a specific part of the architecture proposed: the part which makes use of blockchains as a technical solution, with recognized advantages in maintaining and ensuring the veracity of the data. The use of blockchains is particularly interesting when it is not possible or desirable to have a centralized entity to guarantee the maintenance and veracity of the data. However, on the context of the architecture proposed in this article, there are no major reasons for using blockchains, other than the natural robustness resulting from its redundant and distributed nature. In fact, it is perfectly possible to have a centralized entity, provided it is not any of the companies that supply the transport service, as this would jeopardize the trust in the veracity of the data.

Modum is an enterprise that supplies several solutions to track the transport conditions of merchandise. Being a commercial service, there are not too many details about the implemented solutions or even about their maturity. One of those solutions is ModSense One [21]. It monitors the temperature, and is composed of a physical device (that collects the data) and the background support, including mobile app, blockchain, dashboard and report service. It is an end-to-end solution, where the device is attached to the merchandise in the beginning of the transport, following a protocol that initiates the smart contract with the transportation conditions and relates the transportation ID label (a barcode that is attached to the outside of the packaging) with the device. The device collects data periodically and, as far as possible, ensures that it is available in real time to the final user. Despite being a complete solution, it is ideal for transport companies or companies with logistic activities, in the sense that they monitor their own resources and merchandises, and not to provide service guarantees to third parties. This is evidenced by the technical solutions available, some of which involve the installation of fixed routers (in warehouses or transportation vehicles) to forward the collected data to the cloud, and by the price of the solution and the specialization of equipment and software. ModSense One is not an open solution, designed to be widely adopted, with reduced installation and operation costs, focused on providing guarantees to customers, as intended with the architecture proposed in this article. However, both share many technological options and design considerations.

Roambee [22] is an enterprise that supplies a versatile solution to track merchandise, equipment, machinery and vehicles, among other things, during transport and also in warehouses, or even in store. It is focused on the business market (enterprises) and not on the individual costumer. The solution is based on BeeBeacons, which are monitoring devices that allow, as far as possible, to provide data in real time. According to usage requirements, these devices can acquire the geographical position, temperature, humidity, pressure and even detect some organic gases.

The BeeBeacons communicate, using Bluetooth Low Energy (BLE) [23,24], with a mobile hotspot, a gateway for the BLE devices, that sends data to the cloud (Roambee cloud) using a 3G/4G network. The information is updated every 15 min. Besides collecting data periodically, the BeeBeacons can trigger alert notifications whenever the conditions are out of bounds. Normally the gateway is fixed on the warehouse or on the transport vehicle and is the one that contains the GPS function. The devices are attached to the merchandise but can also be used at fixed positions, like warehouses or refrigerators. If the cellular network is not available, the BeeBeacons record the data on the local storage until they get access to a cellular network. Roambee configured the BeeBeacons to continuously advertise, allowing to read the data directly from a mobile terminal without the need to activate the BeeBeacon to put it in advertising mode. The mobile terminal also behaves as a gateway to forward the data to the cloud. Customers can get reports and have access to dashboards using the Roambee Cloud Portal.

As an architectural solution, Roambee is the closest solution to the one presented in this paper, but the goals, priorities, target market and technical options are quite distinct. Roambee emphasizes the access to the data in real-time and the relationship between time and geographical location, thus being more oriented to logistics management. The structural requirements and technical options were designed with a focus on the customer, which is the logistic operator, and considering that the distances and transport times are not very long. There is no specific concern with the reliability of the data and other issues, like the autonomy of the devices, or investment and operating costs. It doesn’t mean that it is worse, but it was conceived for another situation and goals.

## 3. Technical and Domain Contextualization

### 3.1. The Main Scenario

To understand the issues at stake, it is important to contextualize what is the normal transportation process, what is supposed to be done to supervise the transport conditions and what are the main problems to solve.

Our solution, designed to ensure that the service is provided under the contractual conditions, involves monitoring the transport of the merchandise and providing reliable information about it, namely of any non-conformities that may occur. Above all, there must be a guarantee that the information collected is true and with that information is possible to identify the defaulters and assign responsibilities. From the perspective of the authors, the goal is not to pursue those who do not comply, but to encourage the compliance. If everyone involved has the perception that there is an effective control system, with identification of the responsible for the non-compliances and with imputation of responsibilities, only the ones that truly intend to comply with the transportation conditions will accept to perform it (because the costs of non-compliance are usually much higher than the values charged for the transport service).

It should be noted that, in most transports, several operators are involved, both in the transport itself and in storage. On longer routes, the service can even be provided using several operators, that is, the service is contracted to one operator, who in turn hires others, each responsible for part of the route. It is even normal for a subcontractor to have other subcontractors. Thus, there will certainly be a transfer of merchandise, from operator to operator, and it may even happen that the transfer requires a change of container or means of transport. It is also common for transport to be made through several countries, having to go through several customs, and sometimes being temporarily held in storage places. Nevertheless, the important thing is that the contractual conditions are ensured throughout the entire transport process and that this is proved before all involved.

### 3.2. Technical Options

Conceptually, the solution consists of a monitoring module containing sensors, which collect data on transport conditions, and a processor that does the local control and ensures the export of the data, so that they can be provided to those involved in the transport process.

The most simplified solution would be to extract the data upon delivery to the recipient, and forward such data, in some way, to the involved. If there are non-compliances, the recipient would be immediately informed and would have instant evidence to request the appropriate compensation. The company providing the service, on the other hand, would bear the full responsibility for the non-compliances and would have to pay the losses or trigger the existing insurance. Moreover, the same company would hardly have the means to identify (internally or before subcontractors), those responsible for the non-compliances.

The proposed solution builds on the monitoring of environment variables, or other variables, that are supposed to be kept within certain acceptable ranges, otherwise signaling situations that may have a degrading impact on the transported merchandise. Temperature, humidity, atmospheric pressure, ventilation, light or other forms of radiation are some of the environment variables that might be necessary to monitor. In addition to these, it may also be useful to measure the displacement, inclination, speed and acceleration of the load. These variables are monitored by sensors connected to a microprocessor that, among other things, collects the values, temporarily saves them and, whenever possible, sends them to the cloud.

The merchandise could also be very distinct, in many ways. It may consist of small things to huge containers, requiring different solutions for an effective supervision of the transportation conditions. It could be perishable goods, live animals or very delicate goods. The solution must be usable under any of these situations. Thus, size and portability are the first main requirements, and so the device with all the components should be small.

Also, the transportation of the merchandise could take from a few days to several weeks or even months. During all this period, the monitoring device should be able to monitor the conditions of transportation. Therefore, the energy autonomy is probably the most critical feature and is deeply interrelated with all the other requirements of the solution: portability, for instance, implies the use of batteries; in turn, minimizing the size of the device constrains the battery capacity.

Everything on the device should be idealized to save energy, promoting battery life. As such, both the sensors and microprocessor, and even other required components, must be based on ultra-low power technology. The microprocessor, which is the most energy demanding component, should stay in sleep mode as much time as possible. Moreover, the solution should use digital sensors with configurable trigger limits and/or working ranges. The goal is that the sensors can be continuously measuring the environment, without the support of the microprocessor. Only if some measure goes out of the bounds should the sensor wake the microprocessor up to save/report the non-compliance of the transport conditions. Therefore, only the non-compliant measures are processed, and all the others are ignored. This strategy is critical for the feasibility of a solution.

Once a sensor triggers an interrupt, a suitable action follows. The final goal is that the customer, and the main supplier of the transportation service, be informed about the event behind the interrupt. Conceptually, there are two main options for such notification. One is to pass the data to the players immediately, at the moment when the non-compliance is detected. However, as attractive as it may sound, this approach has several problems, some of which are insurmountable. Energy autonomy could be a critical problem because the available solutions that fit into this approach are quite demanding. The use of technologies like LTE-M or NB-IoT [25,26,27,28,29,30] could be an option, but it is important to consider that the transportation of the merchandise is many times done by boat or airplane, crossing geographical areas where these specialized communication technologies are unavailable, as well as other alternatives like GSM and LTE (or the now-being-deployed 5G). Further, even if they were available, there are other problems to consider. GSM, LTE or 5G communication require too much energy and are expensive for scenarios of continuous or frequent data transmission. To make it worse, it might be necessary to access a global communication provider. The problem, however, is that there are none for GSM/LTE/5G technologies.

Another possibility could be satellite communication, for which there are global providers, and which is available in any part of the globe and very reliable. However, commercial satellite communication was mainly designed to supply telephonic communications for remote areas, where the use of cellular networks is not feasible (like in the middle of the ocean or in the remote wilderness), being essentially focused on providing the service to make and receive voice calls. Data communication is also possible, but the bandwidth is limited, being essentially constrained to text messages, emails and access to light web content (without images or video). Nevertheless, it would be enough for the type of application targeted by our architecture, if not for two issues. The first one is the cost of the devices/technology and of the communication, that are quite expensive compared with the cost of cellular communications (like 4G), therefore making it suitable only if no other solution is available and if the merchandise value justify such option. The second problem is related to the energy necessary to power this technology, which is not compatible with the present requirements.

Other possibilities based on satellite communication might be available on the forthcoming 6G context [31,32,33,34] with the development of non-terrestrial networks (NTN) aiming to promote ubiquitous and high-capacity global connectivity, with direct support for IoT. Or, as result of the initiative of the Third Generation Partnership Project (3GPP), to integrate satellite and terrestrial network infrastructure in the context of 5G. Nevertheless, this will take time, because there are technical challenges to be solved, standards to be met, the need to develop and put into orbit new satellites and even define new models of commercial exploration.

Another option is to save all the collected data on the local data storage of the device (flash memory) and then, when the merchandise is delivered to the customer, extract it from the device, using a sensor network technology with a gateway to the cloud, where the data is made available for the players involved. This option is not only more economical than the always-connected approach, but it is also relatively simple to deploy. The device is parameterized according to the agreed conditions for transportation, attached to the merchandise, and nothing more needs to be done until it is delivered to the recipient, when the data is finally collected. Data can be contextualized into a relative time reference that, on delivery, can be converted to a universal time reference. Indirectly, this permits to identify the operator that was responsible for the transportation when the non-compliance occurred, thus contributing to achieve part of our goals.

In addition to data collection and transmission, other aspects must also be taken into account when designing the solution. One of them, and a critical one for anyone considering adopting the solution, is the acquisition/installation and operating costs. Some merchandise, like art, high precision devices and companion or specially bred animals are quite valuable. But other, more common merchandise, like food, might not be. Therefore, it is quite important that the cost of the monitoring device be reasonable in comparison to the cost of the merchandise. The main reason is that the transportation enterprises will, potentially, need many of these devices, once millions of products are moved every day. Even if not all merchandise requires this kind of monitoring, probably most would benefit from this technology. Therefore, to minimize costs, the devices should be reusable and, if possible, have a significant lifetime, including the battery.

Another concern is the full compliance of the control process by the involved players. As already explained, the transportation may involve many companies and even individual actors. It is very important to promote everyone’s commitment, to ensure the quality of the transportation process. In the authors’ conception, this can be reinforced if a protocol, sustained by facts, is defined to be applied whenever the merchandise passes from one operator to another. The operator who passes the goods wants to be certified that they delivered the merchandise in good condition; and the receiving operator wants to have the guarantee that the merchandise was passed to them in good condition. Everyone is thus aware that will be controlled by whoever passes and receives the goods. This works as a self-control solution that encourages everyone to comply with the agreed conditions. By accepting this beforehand, there will be no exemption from responsibilities in case of non-compliance. In addition to the technical perspective, that aims to achieve an effective solution, the authors are also focused on supplying as many arguments as possible, to promote the effective adoption of the purposed architecture. In this sense, the authors are invested in creating a solution capable of supporting innovative services with high added value.

## 4. The Main Scenario

**Figure 1:**
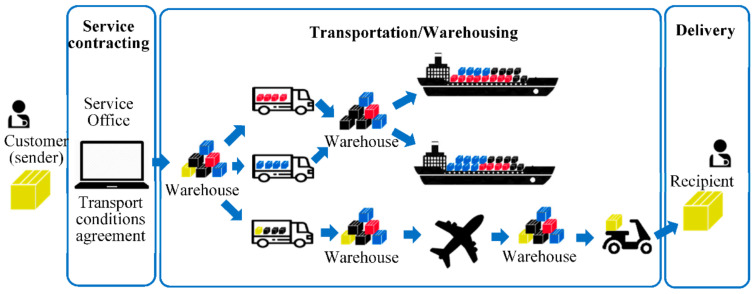
The transportation stages.

Before discussing the details of the proposed architecture, it is important to consider the main stages of the transportation process (see Figure 1). These include:

- The definition of the transport and shipping contract.
- Transporting the merchandise from operator to operator.
- Delivery of the merchandise to the recipient.

The sender is the customer who intends to send the merchandise under certain conditions of transport. He hires the service to a transport company, to which surrenders the merchandise, with the guarantee that it will be transported under the contracted conditions.

In the simplest case, the merchandise is placed directly in the vehicle that takes it to the destination, to be delivered to the recipient. Typically, however, several merchandises will share a single transport, in order to optimize the use of resources. This means that at least an initial storage facility is necessary, to compose the cargo to be transported. This process will be designated by cargo aggregation.

Since the cargo may contain several merchandises, most probably for distinct recipients, located on distinct geographical areas, the inverse process will also happen, meaning that the cargo will have to be disaggregated. Most likely, the aggregation and disaggregation will happen several times during the route between sender and recipient.

The cargo will pass from operator to operator, or from these to logistic warehouses. These are the two main players that we will designate generically by operators. Carriers and warehouses may belong to the same or to distinct companies. Nevertheless, all must know and ensure the contractual conditions. The last carrier is the one that delivers the merchandise to the recipient.

### 4.1. High Level Architecture

The high number of operators that may be involved, their geographical dispersion, and their specific economic and technological contexts, imposes the use of general, universally available, technical solutions.

Smartphones are, nowadays, ubiquitous devices on a global scale, with a broad range of prices and features. Mass production has made them affordable and feature-rich. In terms of business, their use requires low investment, either for those who would have to develop equivalent devices, or for those who would buy them. They feature an easy-to-use user interface, are highly portable, accessible and allow for interesting technical solutions in the scope of the addressed problem. Thus, almost all modern smartphones feature internet access, GPS, Bluetooth Low Energy, a camera and, as already mentioned, the possibility of providing an intuitive user experience. In the context of the work described in this paper, the authors took advantage of all these features to maximize the utility value of the solution.

**Figure 2:**
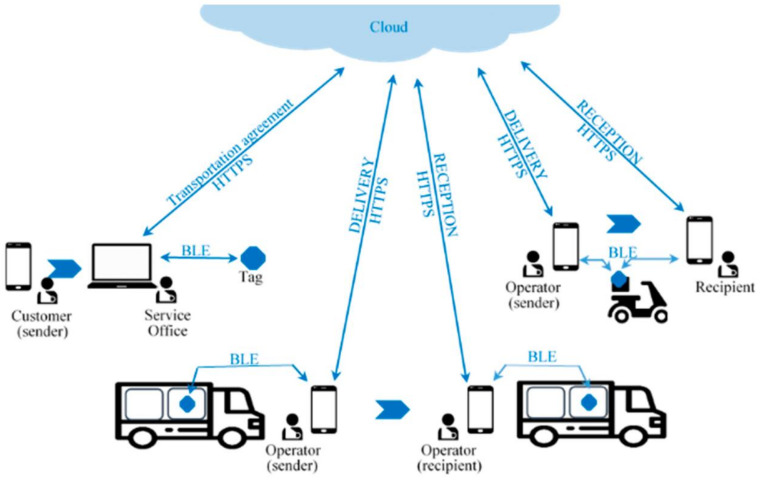
High-level architectural view.

Considering the requirements and the above-mentioned smartphone features, BLE is an attractive technology to use in the development of our architecture. Bluetooth Low Energy is a technological evolution of classic Bluetooth (version 3 and earlier), developed according to requirements that adjusted very well to the needs of IoT, namely allowing communication with very low energy consumption and thus promoting something that is critical for all mobile platforms, which is autonomy [35,36,37,38,39]. Relying on BLE, smartphones and cloud computing, Figure 2 illustrates the high-level architectural view of the proposed solution.

### 4.2. The Monitoring Device

The monitoring of the transport conditions is ensured by a small and lightweight IoT device, designated as Tag, allowing its use in a large number of situations, and achieve a low production and operational cost. The device also comprises a Real Time Clock (RTC), initiated at the beginning of the contract, to control time and validate data.

Most probably, this device is potentially more useful to companies with hundreds or thousands of goods to control. Moreover, since the device follows the goods until they are delivered, the device can only be reused after being returned to a shipping point. It is also expected that the device is subjected to rough conditions and treatments which will contribute to reducing its useful life. Moreover, many will certainly get lost. In other words, it is paramount to keep the production costs of the device as low as possible.

To guarantee a small size and weight, that does not compromise the portability, a non-rechargeable battery powers the device (less components implying lower cost and smaller size). The goal is to reduce the power consumption to the minimal, allowing the device to work for years using a small size battery, like CR2032. To achieve that goal, our specification recommends the use of ultra-low power chips and to maximize the time that they are on sleep mode or equivalent mode (when consumption is so low that it remains in order of few uA or even nA).

To maintain the chip as much as possible on the sleep mode state, all efforts must be done to avoid waking it up with non-useful activities. Instead of periodic and continuous sampling of the transport conditions, our specification recommends only to track the non-compliances. The continuous sampling is an ineffective approach because it requires waking up the chip periodically, most of the times only to confirm what is supposed to be the regular scenario (transport conditions under the agreed values). Instead, in the proposed specification, it is recommended the use of digital sensors to continuously perform the monitoring without the chip involvement, and with the possibility of defining reference limits which, when exceeded, trigger a physical interruption to wake-up the microcontroller. Thus, only in situations of non-compliance of transport conditions, the microcontroller is activated to temporarily contextualize and register the non-compliance. Even considering that the sensor is continuously monitoring the environment, this solution is much more efficient because sensors, especially digital ones, require much less energy than the chip.

In addition, it is also fundamental to minimize the communication periods, in which power consumption, even with low-power technologies, is considerable. The problem is that in order to be able to have connectivity to a BLE device, it must be in advertise mode, which means that there is transmission of an RF signal and, as such, high energy consumption. There are basically two options: keep the device continuously advertising, allowing the establishment of connectivity at any time but reducing drastically battery life; or provide a way to activate the advertise mode with a push button or through RFID. The former requires that the device is physically reachable, and the latter requires bringing the reading device close to the RFID tag. It is also important to consider that RFID technology is not so common in mobiles. In both options, one way to minimize the consumption is to reduce the range of the transmission signal, since the energy consumed is proportional to the radio signal range.

#### Device Types and Topologies

The architecture proposed by the authors considers the following types of Tag devices, covering all the options above discussed for BLE advertising:

- Type A—devices are continuously advertising or advertising periodically, with it being possible to define the power of the transmission signal.
- Type B—the advertise mode is activated by pressing a button and the device stays on that mode for a limited time.

**Figure 3:**
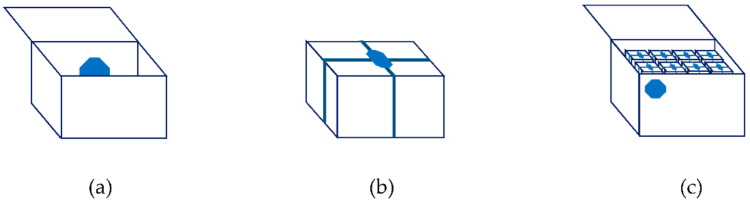
Device usage—topologies. (**a**) Type A device placed inside. (**b**) Type B device placed outside. (**c**) Star network with type B device operating as central (one of the Bluetooth Low Energy (BLE) roles) and placed outside the container and type A or B devices operating as peripherals (another BLE role) placed together with the goods.

The devices can be placed differently when accompanying the merchandises, translating into different network topologies and operational modes. The choice of topology may be dictated by power limitations, like the autonomy of the devices, or operational constrains, as explained below. Figure 3 shows the common usage scenarios for each topology.

On topology (a) there is a single device of type A, placed inside the packaging, working in a continuous advertise mode. This is suitable for the transport of individual goods, with a reduced or medium size volume, or of living animals, where it is not possible to reach the device to activate the advertise. For larger volumes, it is possible to increase the power of the transmission signal or use an external indication of the position of the device inside the packaging. The relationship between journey time, battery lifetime and power of the transmission signal might have to be considered.

On topology (b) there is a single type B device placed within reach so that it is possible to press the button to activate the advertise. With this topology it should be possible to monitor the context variables from the place where the device is placed. It is also applicable for the transport of individual merchandises or non-dangerous living animals.

On topology (c) there are several devices that work together on a star topology. The central node is a type B device that works as a router from the other devices to the mobile network. The devices that monitor the merchandise, that is, the terminal nodes of the star topology, can be of type A or B. The connection among the terminal nodes and the central node is established periodically, at each 15 min, giving the opportunity to the terminal nodes to report any non-compliance to the central node. To put the later in advertise mode, the push button must be pressed. Once connected with the mobile network, it can provide the latest information reported from the related terminal nodes. This topology is adequate whenever there are many goods, it is not feasible to use type A devices on continuous advertise, or the goods are inside a container. The terminal devices are placed with the goods and the central device is placed outside the container or on a place where is possible the manual activation of the advertise.

The decision to use type A or B devices as terminals depends on how the tracking will be done before the aggregation and after the disaggregation of the container. The most common situation is that the transportation of a merchandise starts with topology (a) or (b), and, during the transport process be aggregated and disaggregated several times, switching to topology (c). In the final stage of the route, it is disaggregated, returning to topology (a) or (b). The device type is assigned at service initialization (Section 5.1). The aggregation/disaggregation procedures are explained in Section 5.3.

### 4.3. Bluetooth Low Energy—Services, Characteristics and Access Modes

A brief reference to BLE is provided on this section. BLE has been a privileged technology within the scope of IoT and widely present on mobile phones. It is easy and cheap to implement an application that communicates with BLE devices, to monitor or control them. The mobile phone can even be used as a gateway between BLE and the internet, putting the devices on the net according to the concept of IoT. BLE is also present on tablets, laptops or desktops, which is also relevant for the present work. Additionally, it can be made available as an external module to the chip or embedded in it (SoC—System on a Chip), as is the case of the chip used to validate the architecture (Nordic SemiConductors nRF52832 [40]).

BLE defines two roles: the central and the peripheral. The peripheral defines services for aggregating characteristics. Each characteristic can be available with one or more access modes, such as reading, writing or notification. The central device behaves like a client that connects to a server (peripheral device) and access the characteristics, being the effective communication done by reading and writing in the characteristics. Depending on the processor and of the stack that implements BLE, the write and, eventually, read of a characteristic can trigger an event on the peripheral side. For characteristics available in notification mode, whenever its value changes, there is a notification that triggers an event on the central device. The characteristics are defined within the scope of a service, which can have one or more characteristics. When enabling/disabling a service, the respective characteristics are enabled/disabled.

The devices must include components to signaling their status, allowing users to check whether they are in advertise or connected mode—an LED (Light Emitting Diode) can be used for this purpose. Moreover, Type B devices must also include a button to initiate the advertise mode. In brief, the BLE communication procedure goes through the following steps:

1. The peripheral device is placed in advertise mode that transmits the indication that the device is available, possibly with information about the available services (each service has its own identifier).
2. The central device is placed in scan mode looking for advertise signals. It can be pre-configured to only search for peripherals that advertise certain services.
3. If the peripheral device is found, the central can then request the connection.
4. The connection then takes place, which may involve security procedures, with different levels of implementation—some of which are used in the proposed architecture.
5. The central can read/write in the characteristics available with these types of access or enable the characteristics that can be used on the notification mode.
6. The peripheral device can notify the central device via characteristics of this type (already enabled).
7. The connection between devices can be closed by any of the devices.

The central role is performed by the mobile device (smartphone). It can also be performed by a device operating in (c) mode, as explained at Section 5.3.

## 5. Workflow

### 5.1. Service Initialization

Figure 2 shows the procedures between the actors in the system. The customer/sender contracts the service with the transportation service provider. For this purpose, it defines the transport conditions, which include the limits of the relevant variables and deadlines. The service provider validates the possibility of providing the service, which requires confirming that there are operators to perform all stages of the track under the requested conditions, including deadlines, whether they are carriers, warehouses or others.

If there is an agreement, the process begins, specifying the conditions of transport and descriptive information (of the device, merchandise and transport conditions). Two keys are generated, one that only the sender should have access to (later he will provide that key to the recipient) and another for the service provider. Among other things, these keys are used to retrieve the state and get information about the merchandise, through the portal that gives access to the cloud services. There are two distinct keys, since the information provided to each party (service provider x sender/recipient) is distinct. The service provider has access to all the information supplied to the sender/recipient and to the internal information related to the operators. It is up to the sender to pass the key to the recipient, so that this one can formally receive the merchandise. Non-public identifiers are also generated for the device, merchandise, operators, sender and recipient, as well as two pairs of keys for asymmetric encryption.

A device is initialized through a reset operation, which will immediately activate the configuration mode with the BLE service designed for this purpose. The process stores the following data: the public key of the first pair of asymmetric keys; the private key of the second pair; the identifiers of the device, merchandise, current operator and next operator; transport conditions; descriptive information; and the earliest and later delivery dates (EDD/LDD) on which the merchandise should be delivered to the next operator.

The operating mode and state of the device are also defined, taking into account the way the device is fixed/associated to the merchandise. This state defines the condition of the merchandise in relation to the operator and serves to control the actions that must be available at each moment. In the context of this paper, this state has only two possible values:

DEPOSITED—Indicates that the operator deposited the merchandise to be received by the next operator.
RECEIVED—Indicates that the operator received the merchandise.

**Figure 4:**
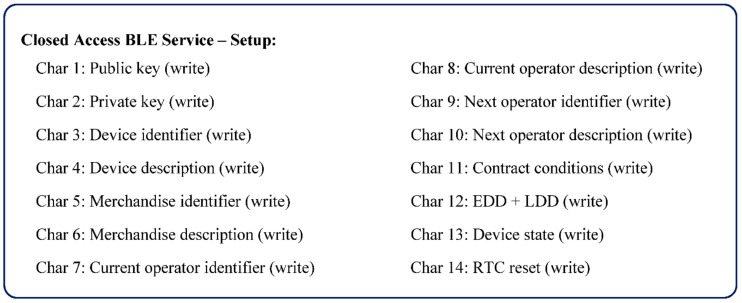
Closed Access BLE Service—Setup.

The RTC of the device is reset and the present GMT+0 universal time is preserved in the cloud registry. Figure 4 shows the BLE characteristics defined for the configuration process, aggregated in what was named as the Closed Access BLE Service—Setup.

In turn, the following data is stored in the cloud: the private key of the first pair of the asymmetric keys; the public key of the second pair; the identification of all operators involved in the transport, by the intervention order, with the respective EDD and LDD, maximum time after deposit that the next operator has to collect the merchandise, and approximate geographical position of the merchandise transfer place; the universal date of the RTC restart and the present state (RECEIVED).

The device is attached to the merchandise in the presence of the customer, with a physical lock that, once applied, triggers the procedure to monitor the merchandise until it is delivered to the recipient. From then on, the device starts to work, collecting data of the non-compliances. If the safety lock is removed ahead of time, the procedure will not be closed or will be closed in an irregular situation. and the operator who officially had the merchandise in his possession will answer for any damages that may occur.

At any time, anyone with the mobile application installed can connect to the device, as long as it is in advertising mode, and access to the descriptive information carried by the device: merchandise, current operator, next operator, EDD/LDD, and state. The descriptive information that is made available can be useful even for those who are not directly involved in the transport of the merchandise, and the fact that it is available does not compromise the security of the system.

### 5.2. Merchandise Transmission

The passage of merchandise between operators is marked by the transition of responsibility between them. It is a critical part of the process because the operator that delivers the merchandise wants to obtain official confirmation that it was delivered and without violations of the transport conditions. In turn, the operator that receives the merchandise wants to be sure that it is in good condition and that no violations of the transport conditions were committed. The idealized protocol aims to provide confidence to both parties, but also to those who may have hired them, whether they are other operators or the final customer.

The process takes place in two stages: the registration of delivery to the next operator, and the registration of receipt by the next operator. The existence of these two procedures results from the fact that a single procedure would require the simultaneous presence of both operators and merchandise. However, it is quite common that an operator deposits the merchandise and performs the delivery register and, only later, the receiver of the merchandise comes to collect and register the reception. During the time in between the delivery registry to the receipt registry, it is still necessary to guarantee the transport conditions and responsibility. It may happen that the conditions are not the ideal, which does not mean that the transport conditions have been necessarily violated. Instead, it means that if that state is kept, the non-compliance will inevitably occur. It may also happen that it is acceptable to violate the transport conditions, but only for limited and well-defined periods. In either case, there is a timeframe for the merchandise to be collected. Otherwise, there will be unconformities in the transport conditions.

**Figure 5:**
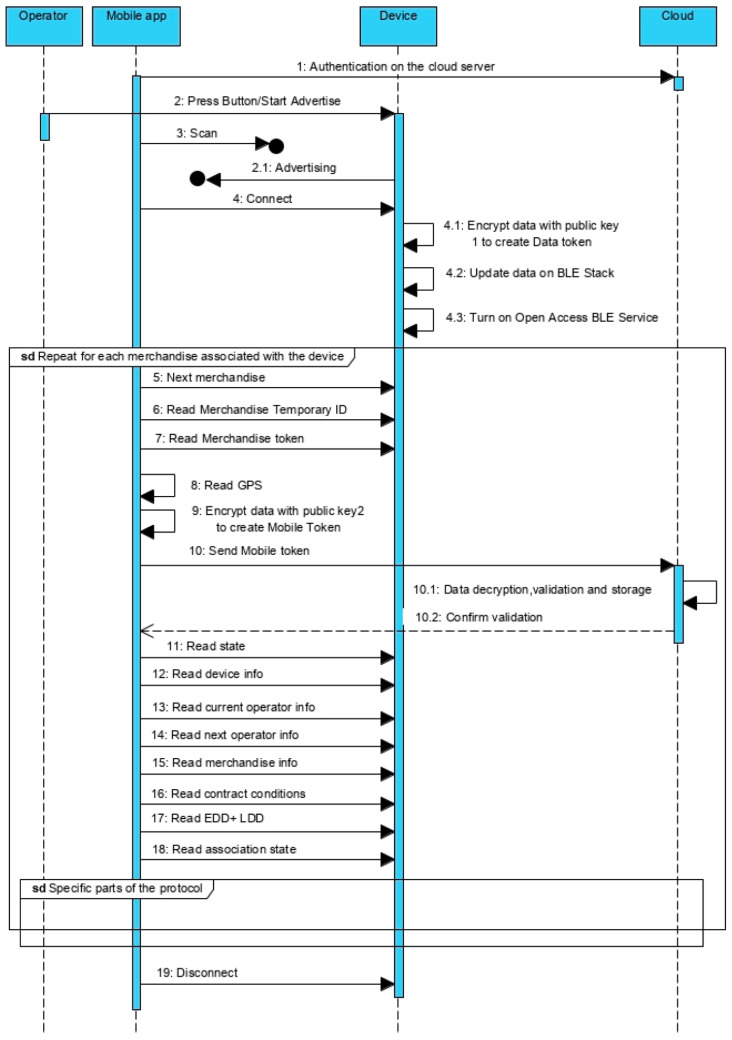
Iteration protocol between mobile app and device.

In both situations—delivery and receipt of the merchandise, the protocol is implemented between the device and the mobile app (Figure 5). It is assumed that, when installing the app, there is an authentication process for the operator (company) and some kind of access token is available on the app. Eventually, it may be necessary to renew the access token regularly.

The common part of the protocol was designed to take advantage of all the possibilities to transfer data from the device to the cloud, even if not directly related to the delivery or reception of the merchandise. It may result from contractual reasons requiring more regular data updates besides the ones associated with the transition of merchandise between operators.

**Figure 6:**
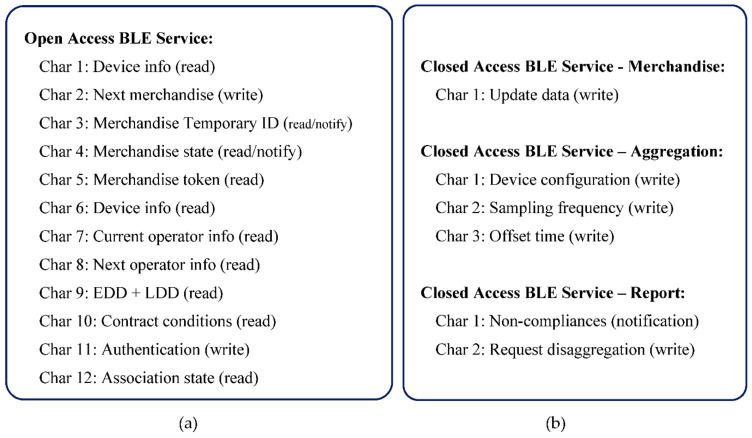
BLE Services API. (**a**) Open Access BLE Service. (**b**) Closed Access BLE Services.

Despite the mobile application being able to make the connection between device and server, the protocol is designed to minimize the information that the mobile device accesses, including information that can be used to vitiate data. To start the protocol, the device should be on advertise mode to allow the connection from the mobile. Type A devices are continuously advertising, but type B and C are not (it is necessary to push the advertise button). Once connected, the device turns on the Open Access BLE Service, that contains ten *read* characteristics, two of type notify, and two others of type write, as defined on Figure 6a. A brief description of these characteristics follows:

- Characteristic 1: Device info (read)—Supplies information about the device to which the mobile is connected. It is a representative characteristic, which in an applied context, may require to be broken down into several, depending on the information to be made available about the device.
- Characteristic 2: Next merchandise (write)—it implements an iterator to access merchandise information and token, one by one.
- Characteristic 3: Merchandise Temporary ID (read)—Supplies the merchandise temporary identifier.
- Characteristic 4: Merchandise state (read)—Supplies the state of the merchandise (DEPOSITED/RECEIVED).
- Characteristic 5: Merchandise token (read)—Allows to read the Merchandise Token pointed by the iterator, including the Data Token (non-compliance measures).
- Characteristic 6–10: These characteristics allow to read information about a merchandise, namely information about: the device attached to the merchandise, current operator, next operator (if exist), Earlier and Later Deliver Dates and contract conditions. On topologies (a) and (b), the device information is the same of the characteristic one.
- Characteristic 11: Authentication (write)—Allows to write the authentication token to activate the Closed Access BLE Services. The token is requested using the mobile app, for operations such as associate/dissociate merchandise to a container, or confirm the receipt of merchandise. Based on the requested operation, it is returned a token that, once authenticated, will turn on the related BLE service.
- Characteristic 12: Used to get the association state among central and terminal devices on type (c) star topologies (see Section 5.3).

Some BLE implementations do not trigger an event on the read-access operations, returning the value that is on the variable associated to the characteristic, which may not be the most up-to-date value. For these BLE implementations, it is necessary to previously guarantee that the associated variables are updated. On the present protocol, the update procedure could be triggered on the connection event (Figure 5—message 4.1).

On type (c) topologies, more than one merchandise is associated to the central device. The terminal devices periodically supply information to the central device and the smartphone is used to get information from the central device about the merchandises. For this reason, an iteration solution is supplied, namely, the Next merchandise (Figure 6a—characteristic 2). Whenever it is called, it passes to the next merchandise information record. The first time it points to the first record and, after the last record, it will produce an empty record (the following requested information will be empty). After the connection is established and the Open Access BLE Service is turned on, the mobile device will initiate the iterator (Figure 5—message 5) to process, one by one, all the merchandises associated to the device (devices on type (a)/(b)). Topologies are associated to a single merchandise, but devices on type (c) topologies can be associated to many merchandises).

Taking advantage of the eventual opportunity to send information to the cloud server, for each merchandise, the mobile apps get from the device the correspondent token (Figure 5—message 7). The Merchandise token is produced using an asymmetrical encryption over a concatenation of the identifiers of device, merchandise, current operator, next operator and a temporary identifier of the merchandise. The last one is created by the device, for local and temporary use, to be supplied to the smartphone, avoiding exposing the system merchandise identifier used on the cloud server/device. It is with this temporary identifier that the mobile app references the merchandise on the iterations with device and cloud server. On the Merchandise token generation is also included the elapsed time since the start of the track, the data related with the non-compliances of the transport conditions and respective collection time.

The encryption is done using the public key generated when the transport service was contracted. The public key follows the device and the private key is associated to the merchandise information on the cloud server.

After receiving the Merchandise token, the mobile app will associate with it the GPS coordinates (Figure 5—message 8) and encrypt everything again using a second public key (Figure 5—message 9), producing the Mobile token that is sent to the central server in the cloud (Figure 5—message 10). On the server, the data is decrypted using the private key, validated and stored. The validation is done matching the values of the device (device, current operator and next operator identifiers) with the values on the server, plus checking if the elapsed time is approximately equal to the difference between the current time and the time recorded when the contract was done. It is also possible to use the GPS coordinates on the validation. Once confirmed that it is a real device, the data is accepted and stored, namely the report of non-compliances.

The mobile app can also request the merchandise temporary identifier (Figure 5—message 6) and information about the device, current operator, next operator, merchandise, contracted conditions and earlier and latest delivery time. This information is used by the mobile app to list the information about the merchandise.

#### 5.2.1. Merchandise Deliver Protocol

**Figure 7:**
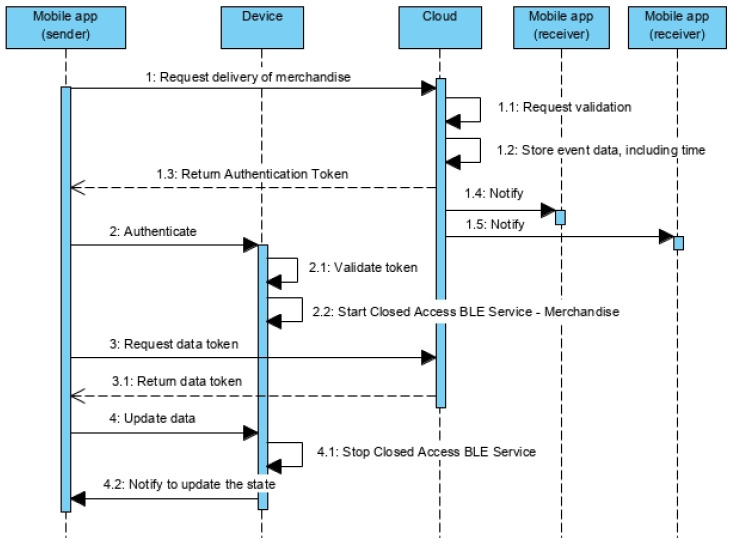
Merchandise deliver protocol.

The merchandise deliver protocol is a specific part of the iteration protocol (Figure 5) between mobile app and device, executed by the operator who wants to deliver the merchandise to the next operator (Figure 7). The operator, using the mobile app, chooses the merchandise and requests the delivery, sending the request to the cloud server and passing as parameter the merchandise temporary identifier (Figure 7—message 1). Notice that the option to request the delivery is only available if the merchandise is stated as RECEIVED.

The cloud server knows the identification of who is requesting the delivery and knows who the current operator is. If both match, the merchandise is marked as DEPOSITED. Besides that, the service generates an Authentication token and encrypts symmetrically the code of the request, the elapsed time since the beginning of the transport (registered on the cloud) and the merchandise identification. The token is passed to the mobile app (Figure 7—message 1.3), that uses it to gain access to the Closed Access BLE Services (characteristic eleven of the Open Access BLE Service) (Figure 7—message 2). The device will decrypt the Authentication token (Figure 7—message 2.1), compare the merchandise temporary identifiers, and also the elapsed time with the Real Time Clock of the device (the comparison accepts a margin of error). If they match, the authentication request is accepted, and the required Close Access BLE Service is turned on (Figure 7—message 2.2).

After that, the mobile app requests the Data token to the cloud server (Figure 7—message 3), passing once more the merchandise temporary identifier. The Data token produced is encrypted symmetrically the merchandise temporary identifier and new state (DEPOSITED). This token is used by the mobile app to update the state of the device (Figure 7—message 4). Once done, the Closed Access BLE service is disabled (Figure 7—message 4.1) and, since the state changed and this is a notification characteristic, the mobile app will be notified to update the state (Figure 7—message 4.2), disabling the option to deliver the merchandise and activate the option to receive it.

Meanwhile, the cloud server also sends a push notification to the mobile phone of the next operator (the merchandise receiver) (Figure 7—message 1.4 and 1.5). The notification will contain the maximum time for the next operator to collect the merchandise, status of the merchandise and other relevant information.

The next operator does not represent a single person, but all the collaborators of the company registered as being able to receive merchandises. Thus, the push notification is sent to all these collaborators (Figure 7—message 1.4 and 1.5).

#### 5.2.2. Merchandise Receipt Protocol

**Figure 8:**
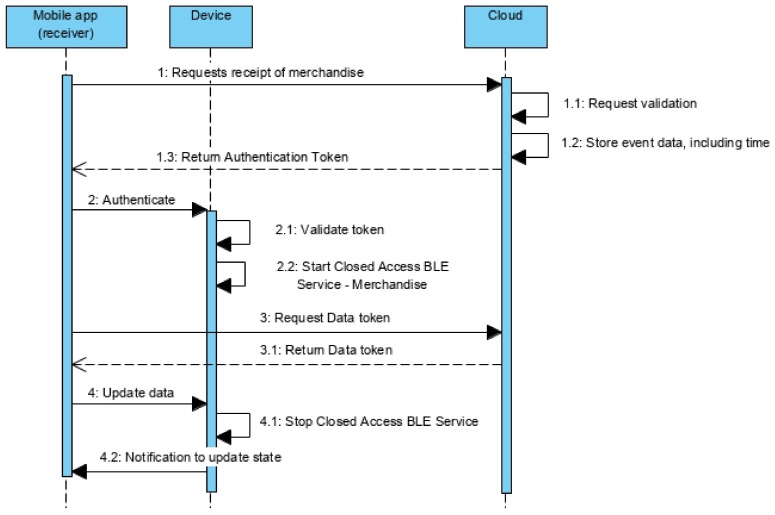
Merchandise receipt protocol.

The merchandise receipt protocol is executed after the iteration protocol (Figure 5) by the operator who wants to formally make the reception of the merchandise. From the smartphone, the operator chooses the merchandise and requests the reception, sending to the cloud server the merchandise temporary identifier (Figure 8—message 1). Notice that the reception option is only available if the state of the merchandise is DEPOSITED.

The authentication procedure is similar to the delivery protocol. Once the Closed Access BLE Services are available, the mobile app requests the Data token to the cloud server (Figure 8—message 3), passing once more the merchandise temporary identifier. The Data token is produced encrypt symmetrically the merchandise temporary identifier, the next operator identifier, the associated info and the maximum time to collect the merchandise after the next delivery. This token is used to update the state of the device (Figure 8—message 4). Once done, the Closed Access BLE service is disabled and the mobile app is notified by the device to update the state (Figure 8—message 4.2), disabling the option to receive the merchandise and enabling the option to deliver the merchandise.

### 5.3. Merchandise Aggregation and Disaggregation

The aggregation of merchandise in a single container is a common practice. It is important to remember that the devices should not be removed from the merchandise. Otherwise, they might not be able to correctly evaluate the transport conditions. That way, whenever the merchandise is added to a container, the device will stay associated to the merchandise and, as consequence, it will go inside the container too. This scenario poses several obstacles and challenges:

- Inside a container there might exist several monitoring devices, associated to merchandises with distinct transport requirements.
- Getting access to the merchandise inside the container might not be easy or even possible, without extra work or without jeopardizing the remaining goods.
- Type B devices probably will not be accessible to activate the advertise mode, which means that it may not be possible to collect the data related with the non-compliances, until the merchandise is removed from the container—too late to restore the optimal transport conditions and/or maybe to save the merchandise.
- It may not even be possible to access type A devices from outside the container, using a simple smartphone.
- If there are many devices inside the container, it might not be easy to access a specific one.

The next sections introduce the specific parts of the architecture conceived to surpass these obstacles/challenges, by adding the type (c) topology.

#### 5.3.1. Type (c) Topology

**Figure 9:**
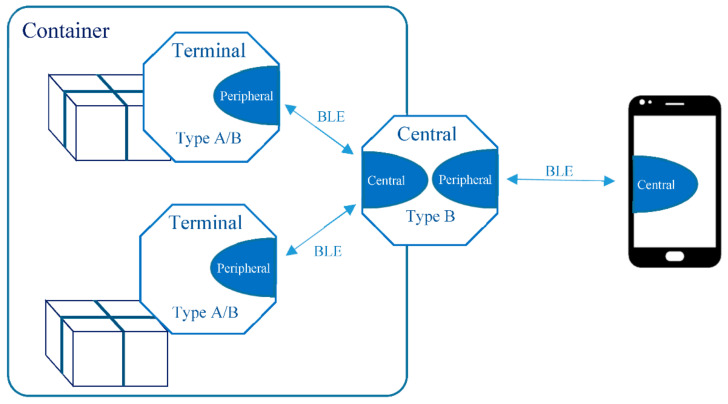
Type (C) topology using a B device as central node and A/B devices as peripherals.

Figure 9 shows the topology of the conceived solution. Outside the container there will be one or more type B devices. Inside there will be the merchandises with the type A and/or B devices. They are connected using a star topology, where a type B device is the central node, and all the others are terminal nodes that communicate with the central node. No communication among terminals is required, other than point-to-point among terminal and central node.

Except for type A devices, which are continuously advertising (awake), all the others are normally sleeping and waking up periodically. If they have non-compliances to report, they send them to the central device. Each non-compliance contains the relative time (time elapsed since the trip started), the identification of the measure and the acquire value. Each report contains the merchandise temporary identifier and the list of non-compliances encrypted using the public key provided when contracting the transport service.

Using the security mode 1 of the BLE standard, level 2, 3 or 4, it is also possible (and desirable) to encrypt the communication channel [41]. Nevertheless, the communication is only possible if each node is on the whitelist of the other. In normal conditions, the whitelist on the central node will have several accepted devices (terminals) and a terminal whitelist will have a single accepted device (the central node).

The Generic Attribute Profile (GATT) establishes how to exchange data over the BLE connection, including the available roles that the interacting devices can adopt. The devices that implement the central role are the ones that scan for the services advertised by those that implement the peripheral role. The BLE services are implemented on the peripherals and each service can contain one or more characteristics. Each characteristic could have one or more access modes. In the present work the modes used are the read, write and notification modes. The read and write modes, when set, allow the central to, respectively, read and write the value of the characteristic. The notification mode enables the automatic notification of the central device, whenever the characteristic value changes on the terminal device.

On the star topology, the terminal nodes implement the peripheral role, and the central node implements both roles, the central to communicate with the terminals, and the peripheral to communicate with the smartphone. By default, the smartphones establish the BLE connections on the role of central.

**Figure 10:**
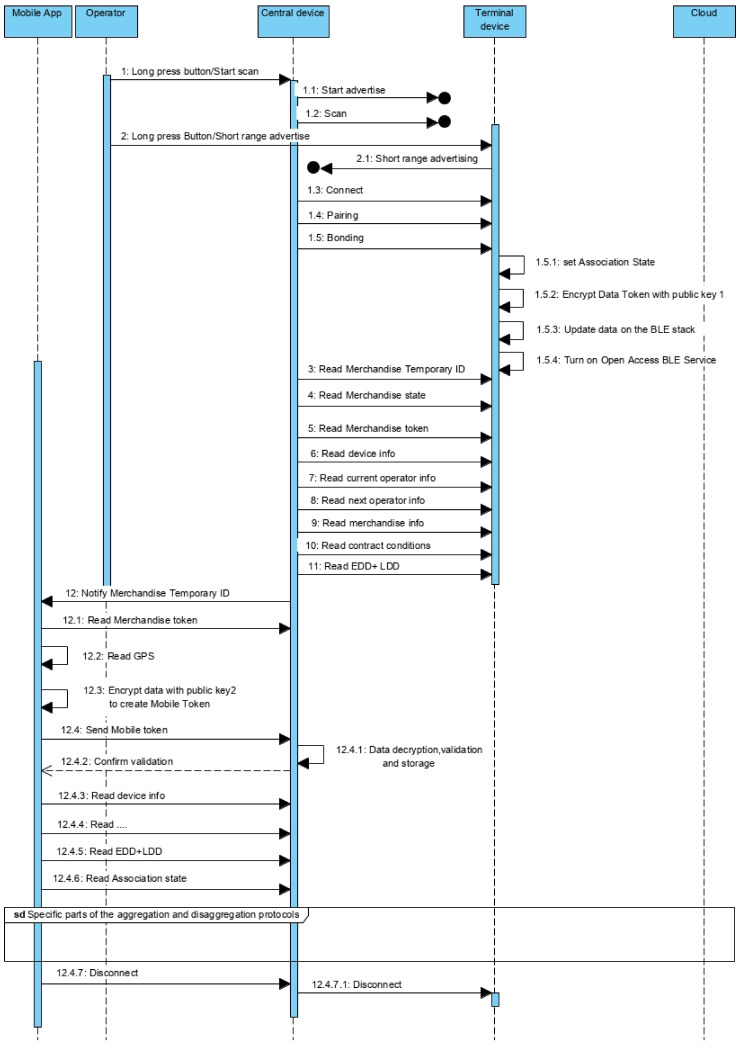
Common part of the aggregation and disaggregation protocols.

Figure 10 represents the common part of the aggregation and disaggregation protocols. Whenever the operator adds a merchandise with a monitor device to a container that supports the star topology, the operator should make the association among devices, making the central node a front-end, accessible from the outside of the container, that reports information collected from the terminals inside the container.

The association is done activating the low range advertise mode on the peripheral (Figure 10—message 2). Controlling the time the button is pressed, it is possible to activate the device for different behaviors (simple click activates the advertise function, long click activates the advertise function in low range mode, very long click resets the device).

With low-range advertise, the radio signal is propagated only for short distances (it can be configured to operate from a few centimeters to a few meters). In this way, the connection between devices is only possible when they are close. Typically, the central node will be fixed somewhere outside the container and the terminal is fixed on the merchandise. The operator will have to bring the merchandise closer to the central device. Previously, it is necessary to activate the scan mode on the central and the low range advertise on the terminal.

The BLE implementation should be prepared to automatically start the pairing and bonding procedure, exchanging the necessary keys to establish a secure communication channel (Figure 10—message 1.4 and 1.5). The approach used is the Just Works, without an explicit authentication, where the Short-Term Keys are exchanged as plain text (without any channel encryption), being susceptible to man-in-the-middle attacks. But by forcing the pairing to take place only when the devices are close reduces the possibilities for such type of attack. On the other hand, the relevant information is encrypted before being sent, so encrypting the communication channel is an extra security. The BLE standard provides more safer solutions, like Passkey Display, that requires more complex user interfaces (LCD and/or keyboard), or Out of Band (OOB) that uses technologies like NFC to implement a safer authentication.

After the bonding, the central node sets the association state characteristic to true if the terminal is already in its whitelist. Otherwise, the characteristic is set to false (Figure 10—message 1.5.1). This state signals if the merchandise is already associated to the container, allowing the operator to request the disaggregation; or, if is not associated, allowing the operator to request the aggregation. On the next stage, the central node reads the information available on the terminal, mainly focused on the merchandise (Figure 10—from message 3 to 11). It starts by the Temporary ID used to index all the data related with the merchandise. Then it reads the Merchandise token, including the Data token with the existent non-compliances. It also reads descriptive information to supply to the operators, including information about device, the self-merchandise, current and next operators, contract conditions, LDD and EDD.

Once all the values are read, the central device triggers a notification to the mobile app (Figure 10—message 12). That notification is a sign for the mobile app to read the Merchandise token, including the Data Token (Figure 10—message 12.1), and implement the same procedure used on the iteration protocol between mobile app and device (Figure 5), aiming to validate on the cloud service the information received from the central (that came from the terminal device) (Figure 10—from message 12.1 to 12.4.6).

Once validated, the mobile app reads the remaining information to display it on the context of the container, including the association state (between terminal and central) (Figure 10—message 12.4.6) to enable one of the request options: merchandise aggregation or disaggregation. The next stage is specific of the aggregation or disaggregation protocols (Section 5.3.2 and Section 5.3.3).

Notice that the information exchange procedure between terminal and central, and between this one and the mobile app, are purposefully similar, normalizing the behavior, the implementation and minimizing the number of BLE services and characteristics.

#### 5.3.2. Aggregation Protocol

**Figure 11:**
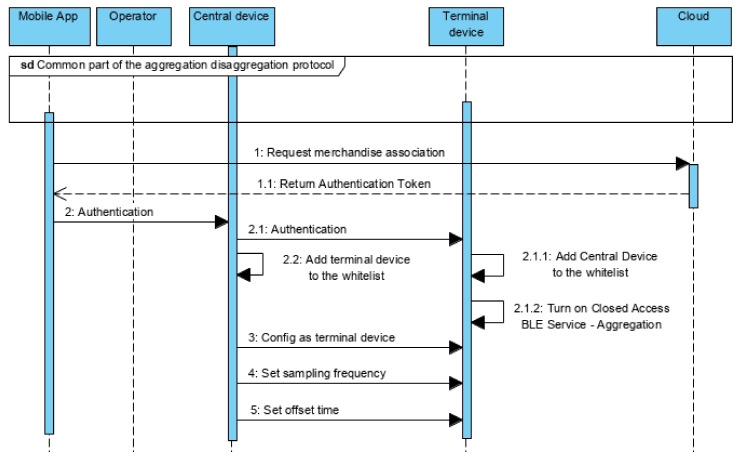
Aggregation protocol.

If the terminal is not part of the whitelist of the central, the association state will be false and the mobile app will enable the option to aggregate the terminal to the central, which means associating the merchandise to the container (Figure 11). If this option is selected, the mobile app sends an aggregation request to the cloud. If the state and information is consistent, the cloud service will reply by sending the Authentication token (see Section 5.2.1).

The Authentication token is used for the mobile app authentication on the central device and activation of the Closed Access BLE Service—Aggregation (Figure 6b). Once authenticated (Figure 11—message 2.1), the central adds the terminal to its whitelist (Figure 11—message 2.2) and the terminal do the same relatively to the central device (Figure 11—message 2.1.1). This will later allow connectivity between devices. With the Closed Access BLE Services enabled (Figure 11—message 2.1.1), the central will configure the type A/B device to work as terminal on the star topology (Figure 11—message 3).

During the transport period in which the merchandise is in the container, both devices will establish the connection periodically. Besides the time interval (Figure 11—message 4), the central should inform the terminal when the first connection starts (Figure 11—message 5). This time offset is computed considering the time from the present moment to the beginning of the next connection period, the number of terminals hold on the whitelist of the central device, the number of simultaneous connections accepted by the central and the relative position of the terminal on the whitelist—the goal is to prevent that all terminals try to request connection simultaneously promoting access conflicts, which would lead to more connection attempts and higher energy expenditure.

The terminal gets that data and configures itself to wake up periodically and establish the connection with the central.

#### 5.3.3. Disaggregation Protocol

**Figure 12:**
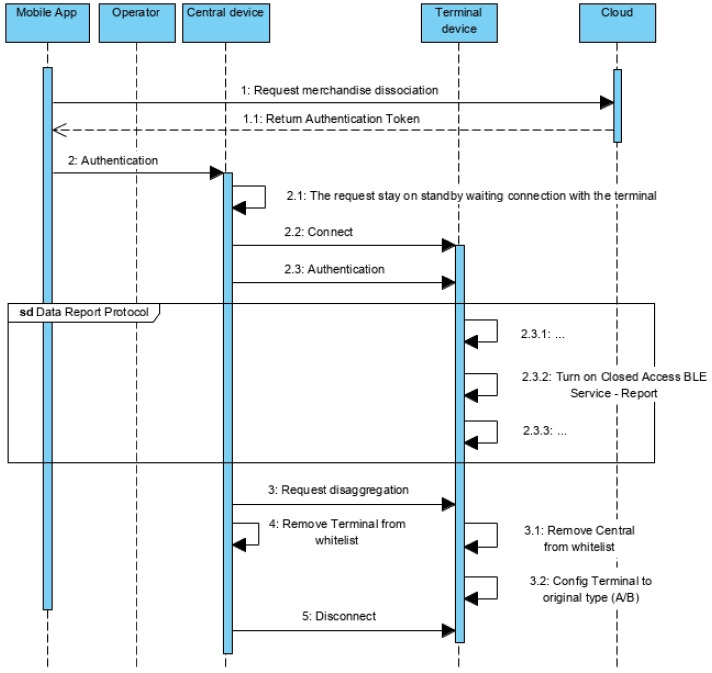
Disaggregation protocol.

If a terminal already belongs to the whitelist of the central, the value of the association state characteristic will be true and the mobile app will enable the option to disaggregate this terminal from the central, which means dissociating the merchandise from the container (Figure 12).

Once more, if the operator selects the disaggregation option, the mobile app request to the cloud server the Authentication token (Figure 12—message 1) that will be used for authentication on the central device (Figure 12—message 2).

Since there is not synchronous communication between central and terminal devices (such conditions are guaranteed on the aggregation, but not on the disaggregation procedure), the central device waits until it gets connected with the terminal, withholding the disaggregation request (Figure 12—message 2.1). Once connected (Figure 12—message 2.1), it authenticates itself on the terminal device (Figure 12—message 2.3), turning on the Closed Access BLE Service—Report (Figure 12—message 2.3.2 and Figure 6b). This service is used to report noncompliance to the central (Section 5.3.4), but also allows the disaggregation procedure, which is initialized at the request of the central (Figure 12—message 3). Consequently, the terminal removes the central from its whitelist (Figure 12—message 3.1) and returns to its initial configuration (Figure 12—message 3.2). For its part, the central removes the terminal from its whitelist (Figure 12—message 4), concluding the disaggregation procedure (Figure 12—message 5).

#### 5.3.4. Report Protocol between Terminal Devices and Central

**Figure 13:**
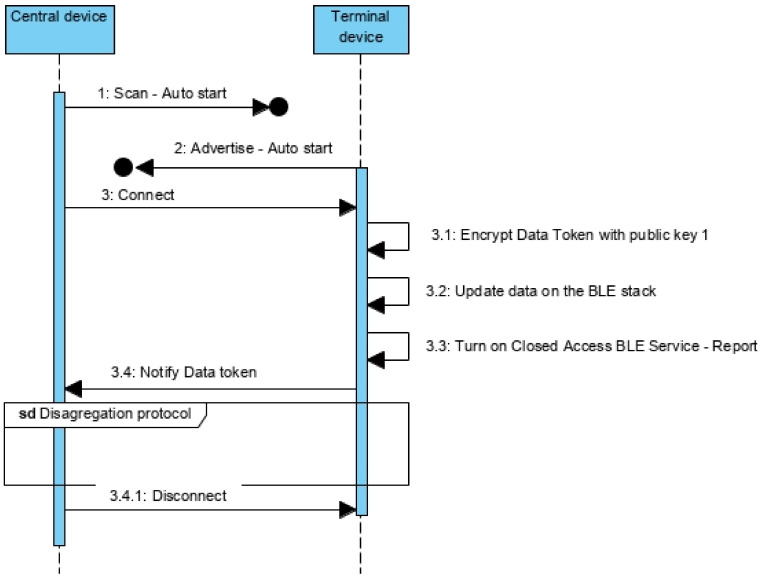
Report protocol for the terminal devices reports nonconformities to the central.

Figure 13 represents the protocol for non-compliances report from the terminals to the central device during the transport period in which the merchandise is in the container. The devices are synchronized during the aggregation protocol as explained in Section 5.3.2. Whenever the central wakes up, it starts scanning for terminal nodes (Figure 13—message 1) that contain the Closed Access BLE Service—Report (Figure 6b) and that belong to the whitelist of the central device. The terminals wake up during the time interval in which the central is available and automatically start to advertise (normal range) (Figure 13—message 2). When the central establishes the connection with the terminal (Figure 13—message 3), it dispatches the creation of the Data token encrypting the non-compliances (Figure 13—message 3.1). The Data token is concatenated to the Merchandise Temporary ID and both are sent as a notification request to the central (Figure 13—from message 3.2 to 3.4). The central adds this information to the local dictionary associating it to the key defined by the Merchandise Temporary ID.

### 5.4. Deliver of Merchandise

The delivery of the merchandise is done in a similar way to the transmission between operators, but now the intervenients are the operator who has the merchandise and the final recipient. Effectively, this is the formal acceptance of the merchandise and the update of the records that may exist on the device since the last update. The data collected throughout the route and stored in the cloud is available to both the sender and the recipient, including the current update, list of non-compliances of transport conditions, a detailed report of the non-compliances (values, time during which the non-compliance occurred, absolute time), expected delivery date and other information.

The same happens with the company that provided the transport service, but this also has access to who were the direct operators and contracted companies hired to perform the service, thus having the possibility of ascertaining the responsibilities for the non-compliances.

## 6. Discussion, Conclusions and Future Work

The architecture described in this work aims to certify the conditions of transport, registering and contextualize, in time and geographically, any noncompliance that might occur. This architecture is generic and can be applied in many contexts, regardless of the geographical scope and range (regional, national or worldwide). It is designed to simultaneously safeguard the interests of the end customer and of the service providers, allowing the provisioning of a high-value service which, by its characteristics, is absolutely relevant in the current context of global trade.

This architecture has several singularities that make it unique. Starting with the motivation: ensuring compliance with transport conditions in an effective and immutable manner, to all those involved in the process—promoting transparency, liability, quality of service and the safeguarding of the goods and of the interests of the consumers, who are the end payers.

Compared with others, including those presented on this paper, the proposed solution can be effectively applied at intercontinental scale and on scenarios where the transport logistics are done involving many suppliers (the most common scenario). Its conception considered the large distances and times, the existence of different means of transport, the nonexistence of a global and effective communication infrastructure, and the involvement of many operators, which, despite cooperating to ensure a service, belong to different enterprises that might not have the same level of commitment or responsibility.

The solution reached is the result of a well-balanced equation between the requirements of the problem that effectively matter, the available technologies, the way that the transport and logistic enterprises are organized and work, and the solution cost (a critical factor to foster its adoption by the market).

For this, important options had to be considered and firmly supported by a careful and sustained study, given that such options are contradictory to the current trends, clearly present in the analyzed solutions, and less attractive from the point of view of the scientific research. There were some major decisions that definitely influenced the proposed architecture:

- Relegating real-time communication to a non-critical requirement—can be critical for specific contexts and an additional added value, but for the vast majority of situations it is of no practical use.
- Supporting the solution with viable technologies, assuming in a pragmatic way that a communications infrastructure on a global scale, with acceptable usage costs (for the problem in question), is not yet a reality.
- Assuming a pragmatic stance regarding the usefulness of information (considering the goals of the solution) and the moments when it is actually necessary—when the merchandise and the responsibility for it passes from one operator to another.

It is important to highlight that the proposed solution has a clear and immediate identification of who did not comply with the agreed conditions to transport the merchandise. Even without using global communication channels, the architecture presented can continuously monitor the merchandise and report non-compliances in useful time, simply using BLE and conventional smartphones/tablets, working without geographic, temporal or distance restrictions. Furthermore, with the proposed technical solution and technologies, the type of battery chosen (CR2032) can last for years, probably beyond the useful life of the device itself.

The cost of adopting such a solution essentially requires the purchase of monitoring devices by the company providing the transport service. The devices are based on affordable technologies and materials and, judging from the experience that the authors have in developing this type of solutions, it is realistic to estimate that the production cost is below $10. The operators only need to have a conventional smartphone/tablet, with the application installed and with access to data communications. No high-value structural investment is required and even the acquisition of monitoring devices may be gradual.

In operational terms, operators have costs with data communication and protocol operationalization, but such costs are quickly covered with the provision of a high-value service (being able to charge more for it). The company that provides the transportation service to the final customer has, as operational costs, the access to the cloud services, that should be provided by a third party other than the company itself, so as not to jeopardize the integrity of the data and the process.

The customer and other operators are assured that the transport was (or was not) carried out as agreed; and in case of non-compliances, it is easy to identify the defaulters and determine responsibilities. This is particularly relevant when the damage is not immediately identifiable in the merchandise.

The authors believe that the requirements defined for the solution match the needs of the market and that, despite being of interest to those involved, it is designed from the perspective of who actually pays—the customer. Nevertheless, part of the future work will validate whether the defined requirements effectively allow a solution that can be implemented and used in a real context and, if the presented work could be the basis for the creation of an open standard.

The architecture is in an advanced phase of prototyping and submitted to several functional tests and technical validation in controlled and properly characterized and identified scenarios. Several complementary requirements have already been identified, which aim to deal with more specific situations, namely, to provide novel services, some of which are already in the design phase. At present, the authors are specifying the structure of the cloud service, based on blockchains and smart contracts technology [42,43,44,45,46].

It will also be useful to analyze the adequacy of the solution to societal crises (such as those related with the operational logistics of vaccines for COVID [47]), as well as conducting several studies on specific parts of the solution and its implementation (as is the case of the comparative analysis of consumption, lifetime and operating costs for the various topologies proposed, considering alternatives regarding the variables to be monitored). Furthermore, there is still a lot of practical and research work to be done about the procedures related with the contracting of the service and how to validate its viability (in useful time).
